# Aberrant Bodies: An Alternative Metabolic Homeostasis Allowing Survivability?

**DOI:** 10.3390/microorganisms12030495

**Published:** 2024-02-29

**Authors:** Thomas Kozusnik, Simone E. Adams, Gilbert Greub

**Affiliations:** Institute of Microbiology, University Hospital of Lausanne, 1005 Lausanne, Switzerland; thomas.kozusnik@chuv.ch (T.K.); simone.adams@chuv.ch (S.E.A.)

**Keywords:** *Chlamydiae*, aberrant bodies, persistence, iron starvation, interferon-gamma

## Abstract

The *Chlamydiae* phylum is comprised of obligate intracellular bacteria including human pathogens such as *Chlamydia trachomatis* and lesser-known *Chlamydia*-related bacteria like *Waddlia chondrophila* or *Simkania negevensis*. Despite broad differences, these bacteria share a similar development including a persistent state induced using stressors such as immune responses, nutrient starvation, or penicillin introduction. In microbiology, this persistent state is identified by enlarged bacteria, called aberrant bodies, which are unable to divide but are able to survive and resume the developmental cycle upon clearance of the stressor. Clinically, chlamydial persistence is thought to be linked to chronic disease and long-term infections with pathogenic strains. This review aims to share and discuss the latest discoveries made on the little-known mechanisms that take place during stress response. The results indicate that an inter-linked homeostasis between iron and tryptophan is required for effective bacterial proliferation. During stress, *Chlamydiae* attempt to compensate by inducing tight regulations of the tryptophan and iron acquisition operons. These compensations allow bacterial survival but result in the halting of cell division. As cell division is tightly linked to peptidoglycan synthesis and regulation, treatment with β-lactamase inhibitors can also exhibit an aberrant body phenotype.

## 1. Introduction

The bacterial phylum of the *Chlamydiae* is comprised of strictly intracellular bacteria including the well-known human pathogens *Chlamydia trachomatis*, *Chlamydia pneumoniae* and *Chlamydia psittaci*, which are part of the *Chlamydiaceae* family. *C. trachomatis* is one of the most common bacterial-induced sexual infections [1]. While genital infections with *C. trachomatis* (genovariants D to K) are commonly asymptomatic (up to 80% in women), they can lead to genital tract inflammation. In males, complications are limited to urethritis, while in females the more serious infections can lead to pelvic inflammatory diseases, ectopic pregnancy, miscarriage, or tubal infertility [2,3,4]. Ocular infection with *C. trachomatis* genovariants A, B, or C can cause trachoma, a disease which may lead to irreversible blindness over time [5]. Other pathogens, such as *C. psittaci*, *C. pneumoniae*, or *C. abortus*, can provoke potentially deadly lung infections in humans. *C. pneumoniae* is spread through small respiratory droplets; *C. psittaci* and *C. abortus* are less common human pathogens that are zoonotic agents transmitted by birds and ruminants, respectively [6,7].

Also, part of the *Chlamydiae* phylum are the recently discovered families referred to as the *Chlamydia*-related bacteria, which include the *Parachlamydiaceae*, *Waddliaceae*, and *Simkaniaceae*. The *Chlamydia*-like species have been observed in mammalian cells, amoeba, and protists [8]. *Chlamydia*-related bacteria are adaptable to a broader range of hosts than the *Chlamydiaceae*, which are limited complex hosts such as mammals, birds, and reptiles [9]. The genomes of *Chlamydia*-related bacteria are approximately twice as large as those of the *Chlamydiaceae* family, resulting in increased metabolic potential, possibly explaining the broader range of hosts available [10]. Despite these differences, all *Chlamydiae* members share a common biphasic developmental cycle.

The cycle initiates when elementary bodies (EBs), the infectious and non-replicative form of the bacteria (~0.3 µm in diameter), adhere to and enter the host cell via endocytosis. Following entry, EBs surround themselves with a protective endosomal vacuole known as the inclusion. As the developmental cycle progresses, EBs transition to a slightly larger (~1 µm), non-infectious and proliferative form named reticulate bodies (RBs). By hijacking the host cell metabolic pathways, RBs acquire the necessary components for replication. During the mid-cycle of infection, RBs begin to re-differentiate into infectious EBs which are then released into the extracellular matrix either by extrusion of the inclusion, or by host cell lysis [10,11].

During the developmental cycle, RBs may be exposed to stress conditions such as nutrient starvation, antibiotic introduction, or host immune responses. If these stressors are introduced, the bacteria enter into a persistent state of infection marked by the presence of a third form of bacteria called aberrant bodies (ABs). During persistence, ABs stop dividing, become enlarged, and no longer produce infectious progeny [12,13,14,15]. However, this phenomenon can be reversed with the clearance of the stress condition, after which ABs can transition back into RBs and resume the developmental cycle. It is hypothesized that the transition to persistent ABs is linked to the chronic physiopathology caused by chlamydial infections in humans [16]. In this article, we will give a summary on the history of aberrant bodies and updates on chlamydial response to different aberrance-inducing conditions, both in terms of biological persistence but also in terms of morphological changes associated with enlarged non-dividing bacteria with or without DNA replication.

## 2. Historical Overview on the Discovery of Aberrant Bodies

**Figure 1:**
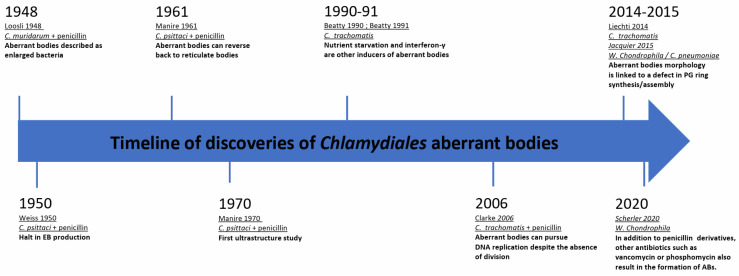
Milestones of chlamydial aberrant bodies research.

Some of the earliest morphological observations of ABs date back to 1948. In vivo, in mice, Loosli et al. observed aberrant growth of *C. muridarum*, which at the time was thought to be a virus, upon introduction of penicillin treatments [17]. Two years later, Weiss et al. made a similar observation during infection of chick embryo cells with *C. psittaci* followed by penicillin treatment. Weiss et al. described the so-called “granules” as greatly enlarged and noted that they could not undergo binary fission after exposure to the antibiotic. Furthermore, it was observed that the RBs on the periphery of the inclusion, which actively divide and closely associate with the inner face of the inclusion membrane, were affected more quickly by the penicillin treatment than those in the center of the inclusion. Ultimately, the bacteria lost their characteristic spherical shape and became irregular “plaques” or ABs [18]. Additionally, Weiss et al. discovered that this persistent state resulted in a halt of EB production. Later investigations of other members of the *Chlamydiaceae* showed that upon penicillin treatment, a similar division and differentiation defect was detected [19,20]. A timeline of these discoveries is presented in Figure 1.

The ability of ABs to reverse the persistent state was unknown until the 1960–1970s. In 1961, Galasso et al. showed that new chlamydial progeny originating from penicillin-induced aberrant bodies of *C. psittaci*-infected HeLa cells were viable following removal of the antibiotic. They found that even if aberrance was induced continuously for over 3 months, drug clearance led to a rapid increase in bacterial titer [21]. Electron microscopy was later used to visualize the morphological structure of the ABs and the bacteria were found to have large, flat sheets of membranes [22].

It was not until the 1990s that nutrient starvation and interferon-γ (IFNγ) treatment were described as additional inducers of persistence in *Chlamydiaceae* [23,24]. Approximately 20 years later, the first publications describing ABs of *Chlamydia*-related bacteria appeared. These studies included *Estrella lausannensis* [25], *Waddlia chondrophila* [26], *Simkania negevensis* [27], *Protochlamydia amoebophila* [28], and *Rhabdochlamydia porcellionis* [29]. Indeed, ABs have been observed in most families in the order *Chlamydiales*. Interestingly, iron starvation and alteration of the peptidoglycan synthesis pathway lead to the observation of aberrantly shaped bacteria in many members of the *Chlamydiae* phylum, as seen in Table 1. Interestingly, in the case of peptidoglycan synthesis disruption, they do not share the same response to drugs. More recently, Scherler et al. demonstrated, using *W*. *chondrophila*, that not only penicillin derivatives act on peptidoglycan biosynthesis. Other antibiotics targeting the peptidoglycan biosynthesis pathways, including vancomycin (targeting the D-ala/D-ala transpeptidase) and phosphomycin, a phosphonic acid antibiotic which targets MurA to inhibit bacterial cell wall biogenesis [27], also yield similar results. While many *Chlamydiaceae* are susceptible to β-lactam antibiotics, some other species, such as *Simkania negevensis*, are completely resistant to them. Even so, *Simkania negevensis* ABs were observed following a high dose of phosphomycin [27].

## 3. The Metabolic Shutdown Induced by Tryptophan Depletion Is Partially Rescued by *Chlamydia*’s Tryptophan Salvaging Pathway through the Preservation of Host Cell c-Myc

Chlamydial persistence originating from IFNγ is one of the most studied stressors due to its clinical relevance [24,57,58]. Interferons are secreted by Th1 cells, cytotoxic T lymphocytes, and NK cells as a part of the innate and adaptive immune response against a wide range of bacteria [59]. During infection of primate cell lines, IFNγ cytokines impact the biosynthesis of indoleamine 2,3 dioxygenase (IDO). IDO catabolizes tryptophan within host cells to reduce bioavailability by producing kynurenines [60]. *C. trachomatis* is auxotrophic for tryptophan synthesis, and without it, replication and re-differentiation are not possible. During infection, some RBs may succumb to a lack of tryptophan availability, but a fraction of them can enter the persistent state due to a mechanism of tryptophan-salvaging from indole [61]. IFNγ, in non-primate mammalian cell lines such as p47 GTPases in mice cells, rather than IDO, is responsible for the tryptophan loss by forcing the pathogen to export tryptophan-rich cytotoxic molecules that will target the p47 GTPases [62].

Survival from tryptophan starvation is possible in *C. trachomatis* genital strains due to the *trpRBA* operon encoded in their genome, with *trpR* being a tryptophan-dependent, auto-repressor of the operon. It is believed that the expression of genes from this operon allows for the conversion of exogenous indole produced by the genital tract microbiota into tryptophan, thereby evading IFNγ-mediated tryptophan starvation. Ocular strains of *C. trachomatis* are more sensitive to the effects of IFNγ, and if a neighboring biovar colonizes the eye, they do not produce indole. Indeed, the ocular strains of *C. trachomatis* have lost their tryptophan synthesis gene function [63]. Evolutionarily, the tryptophan synthesis pathway is weakly conserved among members of the *Chlamydiae*. Some species such as *C. abortus*, *C. pneumoniae*, *C. psittaci*, *Protochlamydia amoebophila*, and *Waddlia chondrophila* have lost all tryptophan-synthesizing genes. *Simkania negevensis* is the only *Chlamydiales* species that encodes the full tryptophan synthesis pathway. The remaining members encode partial operons.

It is interesting to note that despite this high variation in the presence of tryptophan metabolism genes amongst the *Chlamydiae*, there are similarities in the tryptophan content of some proteins. Proteins involved in tryptophan, nucleotide, S-adenosylmethionine, or dicarboxylate transport were shown to have a high percentage of tryptophan residues as do proteins involved in cell division and lipopolysaccharide (LPS) synthesis. A high percentage of tryptophan residues in essential proteins inherently increases *Chlamydiae*’s dependence to the amino acid [62]. However, in the event of tryptophan starvation, it is believed the degradation of previously synthesized tryptophan-rich proteins could help overcome the starvation by maintaining a pool of tryptophan for survival, as it is believed that IDO cannot degrade tryptophan in the bacterial inclusion [61,63].

Most recent studies suggest that IFNγ-induced persistence in *Chlamydiae* is not induced by the activity of IDO, but rather by host cell transcription factor c-Myc [39]. c-Myc is a major polyvalent regulator of cell growth and proliferation and is essential for successful *C. trachomatis* infection. By using a metabolite-uptake assay in an axenic culture, it was demonstrated that *C. trachomatis*’ peptidoglycan synthesis was linked to glutamine uptake. Glutamine plays a key role in EB to RB transition through the stimulation of c-Myc [64]. c-Myc stimulation increases production of glutamine transporters and glutaminases, which are required for chlamydial replication [64]. Upon IFNγ stress, c-Myc levels are depleted concomitantly with TCA cycle intermediary products, leading to chlamydial persistence. Persistence could be rescued with over-expression of c-Myc or by adding TCA intermediates despite the ongoing stress, in cell cultures as well as in organoids derived from human fallopian tubes [39].

In addition to the role of IFNγ on the depletion of c-Myc, tryptophan is involved in the inactivation of glycogen synthase kinase-3 (GSK3β) by phosphorylation. The inactivation of GSK3β prevents c-Myc phosphorylation at threonine 58, which initiates a cascade resulting in its degradation by the proteasome complex [65]. Overall, it seems that IDO allows not only the depletion of an essential tryptophan pool, but that its role also extends to the decrease in a broad range of host metabolism products taken up by the *Chlamydiae* through the indirect degradation of c-Myc [39].

Persistence has also been observed in a murine model of genital infection using the *Chlamydia*-like organism *W. chondrophila*. Fourteen days after infection of mice uterine horns with 10^9^ bacteria, *W. chondrophila* DNA was detectable by RT-qPCR in the liver, spleen, lumbar lymph nodes, and muscles of the mice. Investigation of the IgG response in the mice concluded that IgG2a was the most abundant IgG present, suggesting a Th1-associated humoral response was prominent. Further attempts to re-infect McCoy cell cultures with infected spleen or liver homogenates showed no bacterial replication. While aberrant morphology was not observed, the bacteria were present in a persistent form in McCoy cells infected with liver/spleen homogenates, likely induced by the IFNγ secreted by the stimulated Th1 cells [15].

## 4. The Chlamydial Iron Uptake Operon Is Stimulated upon Iron Starvation and Is Tightly Regulated by Iron and Tryptophan Abundance

In all mammalian cells, free iron is stored in hemoprotein complexes. Building a pool of iron is essential because iron plays a major role in metabolic reactions that require electron exchanges [66]. Storing ferrous [Fe^2+^] and ferric [Fe^3+^] iron in protein complexes allows tight regulation to prevent iron acquisition by potential pathogens. Mammalian ferroxidases are able to convert ferrous iron in the serum to ferric iron. Ferric iron is then able to bind to transferrins with a high affinity. Extracellular iron-transferrin complexes can then bind to transferrin receptors, which are found on the surface of cells, and the entire complex will enter into a target cell through endocytosis [66]. The endocytosis cascade creates a destabilizing, acidic pH in the endosome that frees the ferric iron from the transferrin [66]. The reduction of ferrous iron occurs prior to ferric iron transport in the cytosol. There, it can be used for metabolic processes, such as the TCA cycle or the electron transport chain or it can be stored in a ferritin complex. The free apo-transferrin is then recycled through exocytosis where it can start a new cycle [66]. The current chlamydial iron acquisition model proposes multiple methods for the entry of free iron into the chlamydial inclusion.

One hypothesis is that a proportion of [Fe^2+^] diffuses through the inclusion membrane by passive transport. An active method is thought to happen through slow-recycling iron-transferrin endosomes. These inclusions are Rab11-transferrin-positive and have been observed to be in close vicinity to chlamydial inclusions [67]. These slow-recycling endosomes, which are not immediately sent to degradation by lysosomes and thus have a longer residence time, can transiently fuse to the inclusion membrane, releasing their iron content into the inclusion lumen [67,68]. To date, evidence of this kiss-and-run mechanism is lacking but a similar mechanism was hypothesized in 1998 for phospholipid acquisition [69]. Following fusion, the endosome detaches from the inclusion membrane and enters the classic endosomal recycling pathway. Once within the inclusion lumen, [Fe^2+^] and [Fe^3+^] can access the periplasm of bacteria by passive transport or via siderophore-like receptors, which have not yet been identified [67,68,70]. Transportation into the bacterial cytoplasm is thought to happen through the ABC transporter complex encoded by the ytgABCD operon [71]. YtgC has a C-terminal domain, YtgR, which has been found to be a DtxR-like iron-dependent repressor and is cleaved away from ytgC during infection [72]. This repressor is capable of binding to the promoter of its own operon and likely plays a role in iron homeostasis in all *Chlamydiae* as homologous genes can be found in the genomes of the previously described chlamydial species [73], including chlamydia-related bacteria.

Once iron levels are sufficiently high within the bacterial cell, YgtR also binds the intergenic region (IGR) of the tryptophan operon independently of the TrpR repressor. Thus, there is a need for YtgR to be additionally regulated by tryptophan so that the iron-regulated repression of the tryptophan operon does not occur during tryptophan starvation. Interestingly, by using 2,2-bipyridyl (BPDL), an iron chelator that can bind both [Fe^2+^] and [Fe^3+^], it has been demonstrated that the YtgR repressor also represses the trp operon in *C. trachomatis* in a trp-dependent manner [32]. Due to the rare triple tryptophan motif (WWW) present in the YtgC domain, YgtR levels can be regulated. Regulation occurs when ribosomes fail to read the WWW motif, (i) resulting in a Rho-independent translation termination and (ii) preventing repression of the tryptophan operon, even when iron levels are sufficiently high. YtgC therefore is important in sensing and in regulating both tryptophan and iron metabolism simultaneously [32].

## 5. The Aberrant Body Morphology Induced by Iron Starvation, IFNγ, and β-Lactamase Inhibitors Is Related either to a Defect in Peptidoglycan Ring Assembly or Its Regulation

Persistence induced by IFNγ, iron starvation, or penicillin treatment results in morphologically distinct ABs, which are characterized by their large size in comparison to EBs and RBs [13]. Although currently, there are no precisely defined quantitative parameters of ABs, neither of the AB morphology nor the inclusion size [13,30]. A common point of most of these triggers of persistence is that they affect the peptidoglycan biogenesis pathway. If a stressor is introduced during infection, peptidoglycan synthesis is believed to be down-regulated to limit the release of peptidoglycan compounds that can trigger host cell immunity [74].

Historically, the response to peptidoglycan-disrupting drugs in the *Chlamydiaceae* family remained a mystery, as the presence of a classical peptidoglycan sacculus could not be detected and the role of peptidoglycan was not yet documented. This was referred to as the “chlamydial anomaly” [75]. Later, peptidoglycan was found to be present in members of the *Chlamydiaceae* family [74,76,77]. In 2014, Jacquier et al. and Frandi et al. described in detail the proteins implicated in the division of members of the *Chlamydiae* phylum, including remodeling of peptidoglycan by the AmiA and NlpD amidases [47,78]. Co-temporally, Anthony T. Maurelli enriched the muropeptides belonging to peptidoglycan in *C. trachomatis* and identified their presence through mass spectrometry [77]. Georges Liechti and his team discovered peptidoglycan in small amounts in *C. trachomatis*, thanks to a new cell wall labeling method using d-amino acid dipeptide fluorescent probes, which are incorporated in newly synthetized peptidoglycan [74]. This work convincingly illustrates the polarized division as described in *C. trachomatis* by Abdelrahman in 2016 [79]. Detailed analysis of peptidoglycan degradation products by Greub et al. confirmed the importance of peptidoglycan during division [10,76,80,81]. Interestingly, not all *Chlamydiae* lack a peptidoglycan sacculus. In *Protochlamydia amoebophila*, peptidoglycan could be observed by electron microscopy surrounding the bacteria during replication [82].

In *C. trachomatis*, peptidoglycan has been observed at the divisional plane of the RB which forms the first septal disk. The septal disk expands to form a ring, which then constricts to finalize the division process. In *C. trachomatis*, penicillin binding protein 2 (PBP2) and PBP3 are two regulators which are independently responsible for the peptidoglycan ring expansion and constriction, respectively [83,84]. It has been observed that the volume and thickness of the ring decreases with the number of divisions that have occurred, resulting in smaller bacterial progeny size as infection time progresses. This study correlates well with the description made in 2018 that the transition from RB back to EB occurs after subsequent divisions reduce the chlamydial bacteria size below a certain threshold [85]. The enlarged phenotype of ABs is a result of the absence of division, despite continued bacterial growth. This occurs through the prevention of three different processes, peptidoglycan ring expansion, ring constriction, or peptidoglycan metabolism, as described by Cox et al. [84]. Peptidoglycan ring expansion can be blocked using a PBP2 inhibitor such as mecillinam. Alternatively, peptidoglycan ring constriction can be inhibited by PBP3 inhibitors such as piperacillin. Broad spectrum β-lactamase inhibitors, like penicillin G, are able to target both PBP2 and PBP3 [83,84]. It is likely that a similar mechanism is present in the other members of the *Chlamydiae* phylum, as they all have homologs of PBP2 and PBP3 [73].

Scherler conducted a comparative study of ABs of a distant relative of *C. trachomatis*: *Waddlia chondrophila*. ABs were induced by glycopeptides, penicillins, or iron chelators. There, a threshold of length and area was defined to classify a bacterium as an AB. It was found that treatment of Vero cells with phosphomycin (500 µg/mL), penicillin G (1000 µg/mL), clavulanic acid (900 µg/mL), piperacillin (500 µg/mL), mecillinam (200 µg/mL), deferoxamine (400 µM), and 2,2′ bi-pyridyl (100 µM) resulted in inclusions containing a great majority of ABs compared to RBs. Treatments with MP265 (100 µM), teicoplanin (250 µg/mL), and novobiocin (450 µM) led to more heterogenous mixes of RBs and ABs. The different treatment types also resulted in diversity in AB area, AB length, and number of ABs per inclusion. The treatments also differently impacted the ability of the ABs to replicate DNA. Mecillinam and phosphomycin treatment reduced DNA replication by less than 10-fold while iron-chelating drugs almost completely stopped the process. This study illustrates that variations in the formation of ABs in *Chlamydia*-like organisms results from different response mechanisms based on the stimulus present. This approach assessed the variations in AB size and showed that MP265 induces small aberrant bodies [13]. MP265 is a chemical compound, which binds to MreB to prevent filament formation and is thus critical in cell shaping. If MP265 is added early in the infection, the treated bacteria would exhibit aberrant bodies of similar morphology and size as compared to healthy RBs [13,86].

While penicillins affect division by preventing peptidoglycan ring constriction or expansion, persistence inducers such as iron or tryptophan starvation may instead affect peptidoglycan metabolism by blocking the synthesis of necessary peptidoglycan precursors. Interestingly, bactoprenol, one such peptidoglycan precursor, is synthetized by the methylerythritol phosphate pathway (MEP) which is both iron and pyruvate dependent and widely found in bacteria [87]. The MEP pathway initiates with a pyruvate substrate that goes through several reactions to yield bactoprenol, a carrier module known to transport peptidoglycan monomers into existing peptidoglycan chains that are being synthesized. Pyruvate is also a key molecule during the TCA cycle. Therefore, tryptophan depletion may indirectly prevent the MEP pathway from functioning by downregulating c-Myc, ultimately preventing the synthesis of bactoprenol required for peptidoglycan synthesis. In the MEP pathway, the transition from methyl-D-erythritol-2,4-cyclodiphosphate (MEcPP) to isoprenoid precursors isopentenyl pyrophosphate (IPP) and dimethylallyl pyrophosphate (DMAPP) requires two reactions catalyzed by two enzymes, IspG and IspH, respectively. These enzymes contain iron–sulfur clusters which are obtained from free cytosolic iron. During iron starvation, these enzymes are non-functional, potentially halting peptidoglycan synthesis and thus chlamydial division [88].

## 6. Conclusions/Discussion

**Figure 2:**
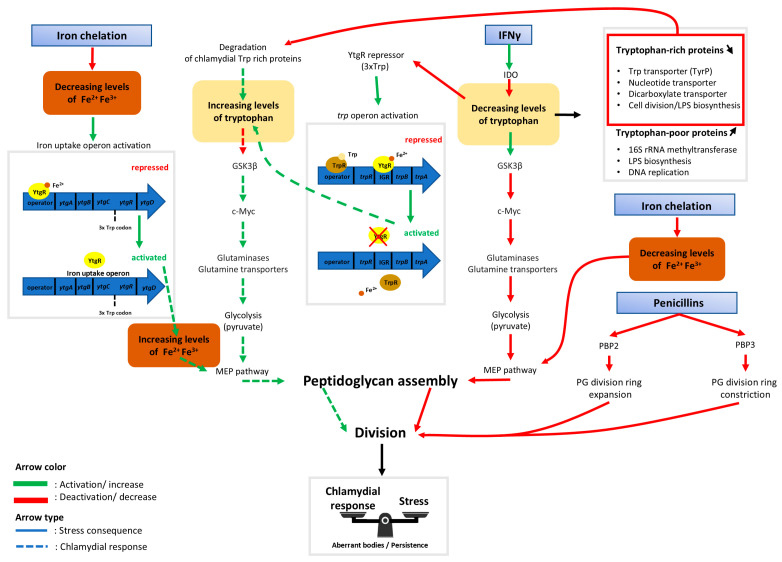
Schematic indicating dynamics between host cell metabolism and the chlamydial response to iron starvation, IFNγ, and antibiotics acting on peptidoglycan biosynthesis. Rectangles with blue gradient represent aberrant body (AB) inducers. Following host cell immune response by interferon gamma (IFNγ), the cytoplasmic tryptophan pool is depleted by the indolamine 2,3-dioxygenase enzyme (IDO) [59]. The absence of tryptophan then activates glycogen synthase kinase-3 beta (GSK3β), an enzyme that initiates host cell c-Myc degradation [39]. The consequence of this is a decreased production of glutaminases and glutamine transporters necessary for the TCA cycle to function. In addition to this reduced function, glycolysis is slowed down resulting in a decrease in pyruvate production [39]. Therefore, there may not be sufficient pyruvate necessary for bactoprenol synthesis by the methylerythritol (MEP) pathway [87]. Bactoprenol is an essential compound of the bacterial peptidoglycan ring, so without it, division is halted, leading to the production of aberrant bodies. If there is a lack of tryptophan (Trp) available, the *trpRBA* operon, which is normally repressed by both TrpR and YtgR, is activated. TrpR repression is released due to the absence of the necessary tryptophan cofactor while YtgR repression stops because there is not enough tryptophan available to assemble the unstable 3xTrp motif in its sequence [32]. To counteract the effects of tryptophan deprivation, tryptophan-rich chlamydial proteins are degraded to refill the pool of bacterial tryptophan, which is sequestered from the host cell and isolated from IDO [62]. During iron stress, proliferation-favorable homeostasis is disrupted. The absence of free ferrous iron prevents the synthesis of peptidoglycan (PG) via the blockage of the MEP pathway due to the lack of two iron-dependent enzymes, IspH and IspG [88]. The *ytgABCD* operon then becomes activated due to the lack of a [Fe^2+^] cofactor which is required for YtgR-mediated repression [32]. While the chlamydial response allows an increase to the pools of tryptophan and of free iron, this compensation might not be sufficient to completely overcome the stress. Phosphomycin and glycopeptide antibiotics such as vancomycin and teicoplanin induce aberrant bodies by affecting directly the peptidoglycan synthesis pathway [13,76]. Penicillin derivatives target penicillin binding proteins (PBP) to prevent the peptidoglycan division ring from expanding or constricting [76]. Together, this may create a new metabolic equilibrium that allows bacterial survival at the cost of proliferation.

Throughout this review, progress in the understanding of ABs induced by tryptophan and iron starvation in the *Chlamydiae* phylum has been shown. The latest discoveries have unveiled the links between the two different types of stressors on the bi-sensitive *ytg*R repressor of the *ytg*ABCD iron operon and the *trp* operon. Repression of these operons is both iron and tryptophan dependent and regulating mechanisms are enacted to prevent a self-sabotaging repression only when tryptophan is depleted. Details of the findings summarized here can be found in Figure 2.

The current hypothesis indicates that, following host cell immune response by IFNγ, the host cell tryptophan pool is depleted by the enzyme IDO. The absence of tryptophan activates GSK3β, an enzyme known to initiate host cell c-Myc degradation. This results in a decrease in the production of glutaminases and glutamine transporters necessary for the TCA cycle to perform optimally. With the halting of the TCA cycle, pyruvate, which is necessary for the bactoprenol synthesis by the MEP pathway, is not produced in a sufficient concentration. Bactoprenol is an essential compound of the peptidoglycan ring and therefore, division is halted, leading to ABs. The persistence of ABs may be a result of their ability to counteract the consequences of stressors. When tryptophan is absent, the *trpRBA* operon, normally repressed by both TrpR and YtgR, is activated. TrpR repression ends due to the absence of a necessary tryptophan co-factor, while YtgR repression stops once there is an insufficient pool of tryptophan to assemble the 3xtrp motif in its sequence. Moreover, degradation of previously produced tryptophan-rich chlamydial proteins refills the tryptophan pool within the inclusion, which is inaccessible to IDO in the host cytoplasm. While the chlamydial response is not robust enough to completely overcome the immune response, it is able to create a metabolic equilibrium to allow bacterial survival.

During iron stress, proliferation-favorable homeostasis is also disrupted. The absence of free ferrous iron may prevent the synthesis of peptidoglycan through the inactivation of iron-dependent enzymes IspH and IspG, thereby blocking the MEP pathway. Peptidoglycan is necessary during division due to its presence in the peptidoglycan division ring, which must expand and then constrict to complete a round of replication. The *ytgABCD* operon is then activated due to the lack of [Fe^2+^] cofactor required for YtgR-mediated repression. Although iron uptake by *Chlamydiae* is high during infection, it is not sufficient to overcome once there is a lack of iron, leading to another inducer of persistence.

β-lactamase inhibitors also induce persistence in *Chlamydiae*. These inhibitors act on the peptidoglycan division ring in a different mechanism to iron stress detailed before. Rather than preventing the synthesis of peptidoglycan, β-lactamase inhibitors affect the regulation of the ring itself by either preventing the expansion of the ring during the initiation of division or the constriction that is necessary to complete division. In each scenario detailed here, the outcome is a termination of cell division characteristic of persistent ABs.

Proposing a general model that would encompass the effect of different stressors for all members of the family is an extremely tedious task due to multiple layers of complexity that occur. One layer is the genomic variation between *Chlamydiaceae* in the *trp* operon. Some *Chlamydiae* genomes, such as *W. chondrophila*, do not contain any known *trp* operon. Even so, by aligning the YtgR sequences of different *Chlamydiae* species, it was shown that *W. chondrophila* does indeed contain the triple-tryptophan motif in its YtgR amino acid sequence that is believed to regulate the repression of the *trp* operon in other *Chlamydiae*. Inversely, *S. negevensis*, which does encode a full *trp* operon, does not harbor this triple-tryptophan regulating sequence in its YtgR repressor [73]. Additionally, both *W. chondrophila* and *S. negevensis* are known to infect a broad range of host cells including protozoa, amoeba, and mammalian cells. Each host has a unique capacity for immune response and iron or tryptophan utilization, adding an additional layer of complexity to modeling the persistence response in these bacterial species.

To further investigate the global regulation of different *Chlamydiae* species, transcriptional studies have been performed under multiple conditions like heat shock, iron starvation, or tryptophan starvation [33,36,55,89,90]. However, comparing the results of the assays has proven to be difficult. For example, RNA sequencing data derived following treatment of cultured cells infected with *W. chondrophila* [55] or *C. trachomatis* [33] with iron chelator BPDL to induce persistence, resulted in dissimilar regulations for most genes. Strikingly, the bacterial stress operon regulated by HrcA was activated for *W. chondrophila*, but not for *C. trachomatis*. Conversely, this operon was activated in persistent *C. trachomatis* during heat shock [36]. Differential triggering of HrcA response between different chlamydial species in the same type of host cell may reflect different susceptibility. These results imply that *Chlamydiae* have a metabolic toolkit optimized for a specific target organism. This aligns with the fact that these intracellular bacteria have a greatly reduced genome, partially resulting from co-evolution with a specific host [91,92]. The importance of host cell compatibility is greatly illustrated in studies where spontaneous ABs were observed without any treatment, as infection in a non-optimal host will increase stress for those bacteria (Table 1).

Additionally, the parameters that have been historically used to characterize ABs separately from EBs or RBs have been described here. Recent evidence suggests that these historic classifications may not encompass all types of ABs which can arise. For example, following treatment of cell cultures infected with either *C. trachomatis* or *W. chondrophila* with MP265, an MreB inhibitor, the bacteria exhibited persistent behavior, but there was no significant increase in the area and length of the cells, depending on the timing of the treatment [13,30]. It would therefore appear that the enlarged bacterial size is not mandatory when peptidoglycan disruption co-occurs with cell wall synthesis inhibition. Given this new finding, it would be prudent to re-examine those previous studies that excluded a persistence phenotype based on size alone to potentially uncover novel insights.

In vitro studies of aberrant bodies generally focus on each stress individually, while their occurrence in vivo is more probably multifactorial. Genomic and metabolomic studies could help assess the level of synergy or incompatibility between chlamydial species and their potential host cells. Moving forward, examining the differential susceptibility of stress from one host cell to another can provide new understanding of chlamydial infection. These new insights could help provide an explanation as to why enlarged ABs have rarely been described in vivo, and why they can appear spontaneously.

## Figures and Tables

**Table 1:** Table referencing research papers describing the presence of aberrant bodies in members of the *Chlamydiae* phylum with different types of stressors.

	Peptido-Glycan Synthesis Inhibitors	Glycopeptide Antibiotics	Iron Starvation	Heat Shock	Co-Infection	Protein Synthesis Inhibitors	Immune-Response	Spontaneous
*Chlamydia trachomatis*	**✓**[30,31]	**✓**[30]	**✓**[30,32,33,34]	**✓**[35,36]	**✓**[37,38]	**✓**[30]	**✓**[14,23,39]	
*Chlamydia* *muridarum*	**✓**[17,40]					**✓**[41]		
*Chlamydia* *suis*	**✓**[42]							**✓**[43]
*Chlamydia psittaci*	**✓**[18,22,44]		**✓**[44]				**✓**[44,45]	**✓**[46]
*Chlamydia pneumoniae*	**✓**[47]		**✓**[48,49]	**✓**[49]			**✓**[49,50]	
*Chlamydia* *pecorum*	**✓**[51]				**✓**[52]			**✓**[53]
*Estrella* *lausannensis*	**✓**[25]							**✓**[54]
*Rhabdochlamydia* *porcellonis*								**✓**[29]
*Waddlia* *chondrophila*	**✓**[13,47]	**✓**[13]	**✓**[13,55]	**✓**unpublished		**✓**[13]	**✓**unpublished	
*Simkania* *negevensis*	**✓**[27]		**✓**[56]			**✓**[56]		

## Data Availability

The unpublished data may be made available, upon reasonable request, from G. Greub, but you may explore more deeply the genomics pathways discussed using our chlamDB database available for free at the following website: https://chlamdb.ch (accessed on 24 February 2024).
