# Correction: Kozusnik et al. Aberrant Bodies: An Alternative Metabolic Homeostasis Allowing Survivability? *Microorganisms* 2024, *12*, 495

**DOI:** 10.3390/microorganisms13122769

**Published:** 2025-12-05

**Authors:** Thomas Kozusnik, Simone E. Adams, Gilbert Greub

**Affiliations:** Institute of Microbiology, University Hospital of Lausanne, 1005 Lausanne, Switzerland; thomas.kozusnik@chuv.ch (T.K.); simone.adams@chuv.ch (S.E.A.)

In the original publication [1], there was a mistake in Figure 2 as published [1]. In the bottom right part of the figure, the “Iron chelation” box points to another box indicating “Increasing levels of Fe^2+^ Fe^3+^” in the published article. This box should instead state “Decreasing levels of Fe^2+^ Fe^3+^”.

The corrected Figure 2 appears below.


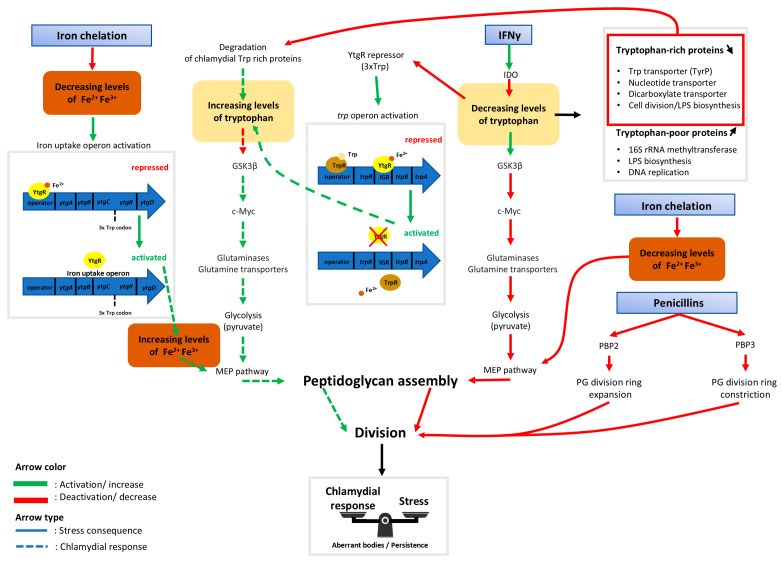


The authors state that the scientific conclusions are unaffected. This correction was approved by the Academic Editor. The original publication has also been updated.

## References

[1] Kozusnik T., Adams S.E., Greub G. (2024). Aberrant Bodies: An Alternative Metabolic Homeostasis Allowing Survivability?. Microorganisms.

